# Supplementary figures and images for: Atribacteria Reproducing over Millions of Years in the Atlantic Abyssal Subseafloor
Source: mBio. 2020 Oct 6;11(5):e01937-20. doi: 10.1128/mBio.01937-20 (PMC7542362; doi:10.1128/mBio.01937-20)

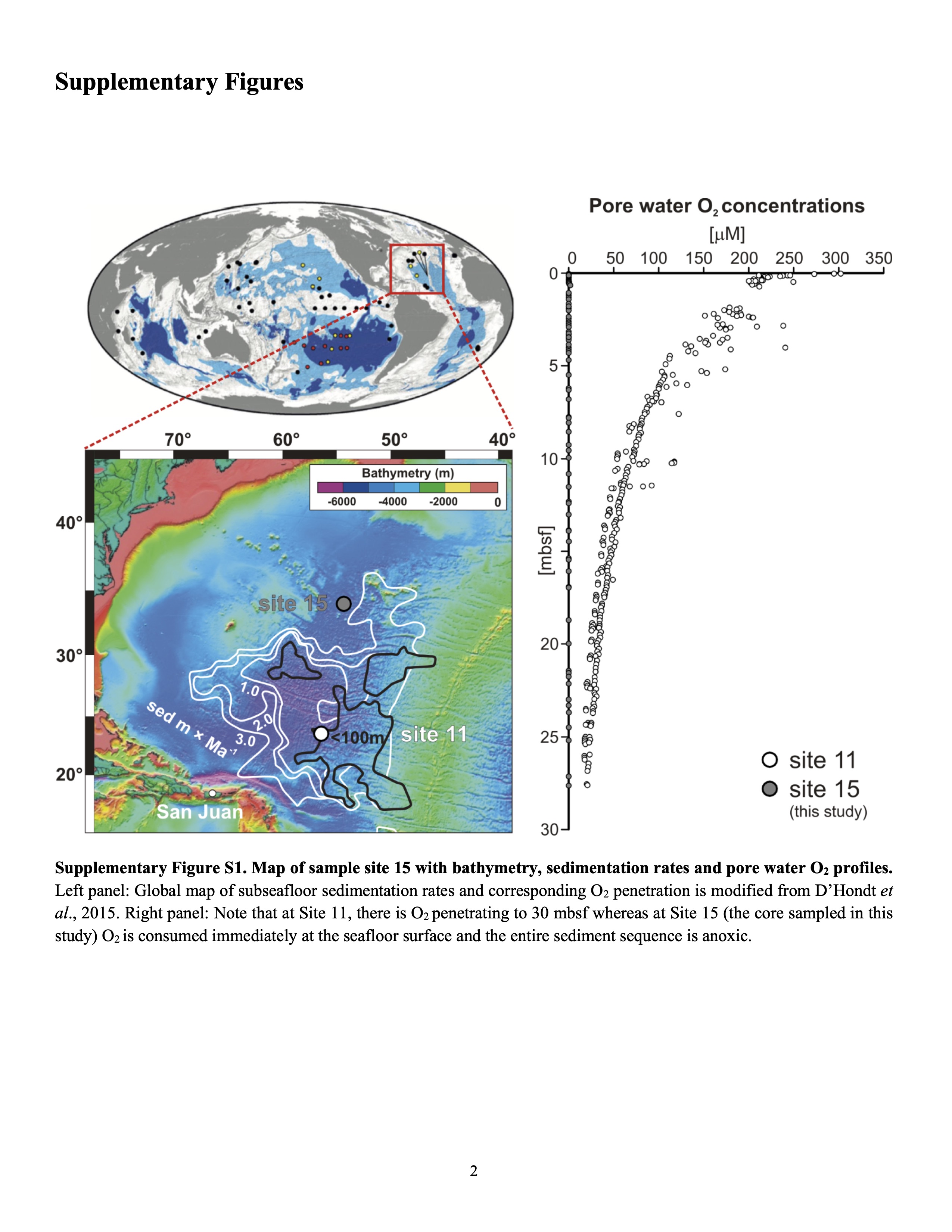

Supplement: FIG S1 [file mBio.01937-20-sf001.jpg]

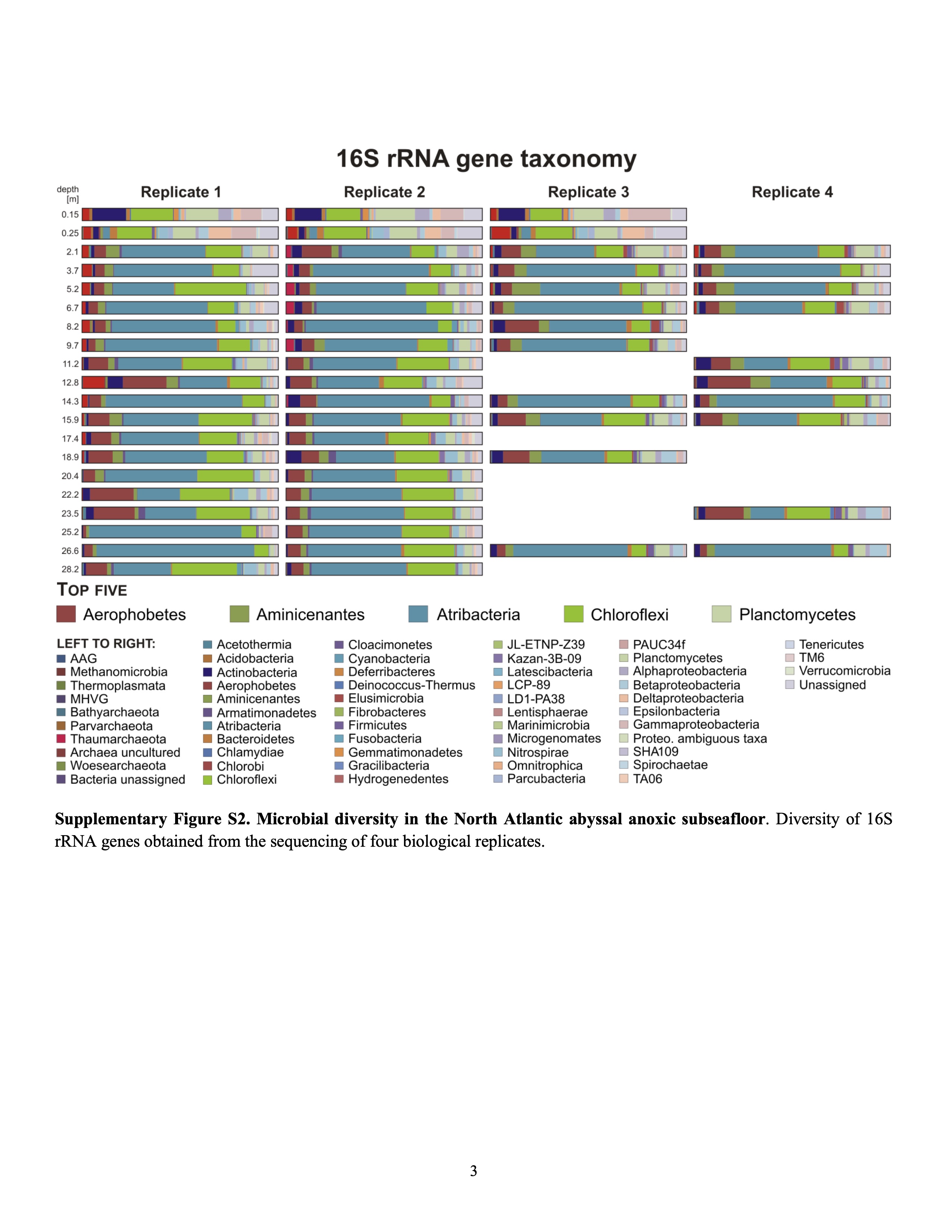

Supplement: FIG S2 [file mBio.01937-20-sf002.jpg]

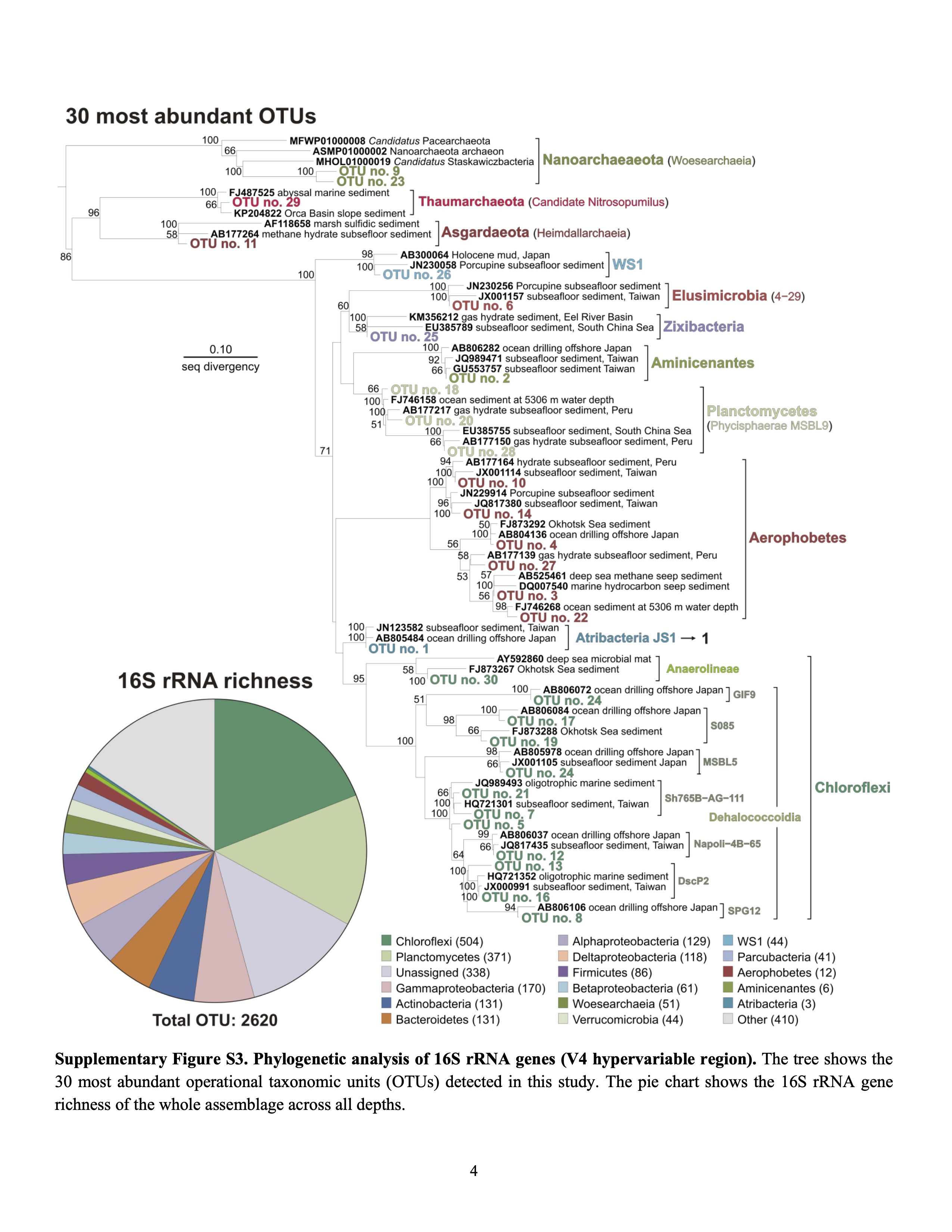

Supplement: FIG S3 [file mBio.01937-20-sf003.jpg]

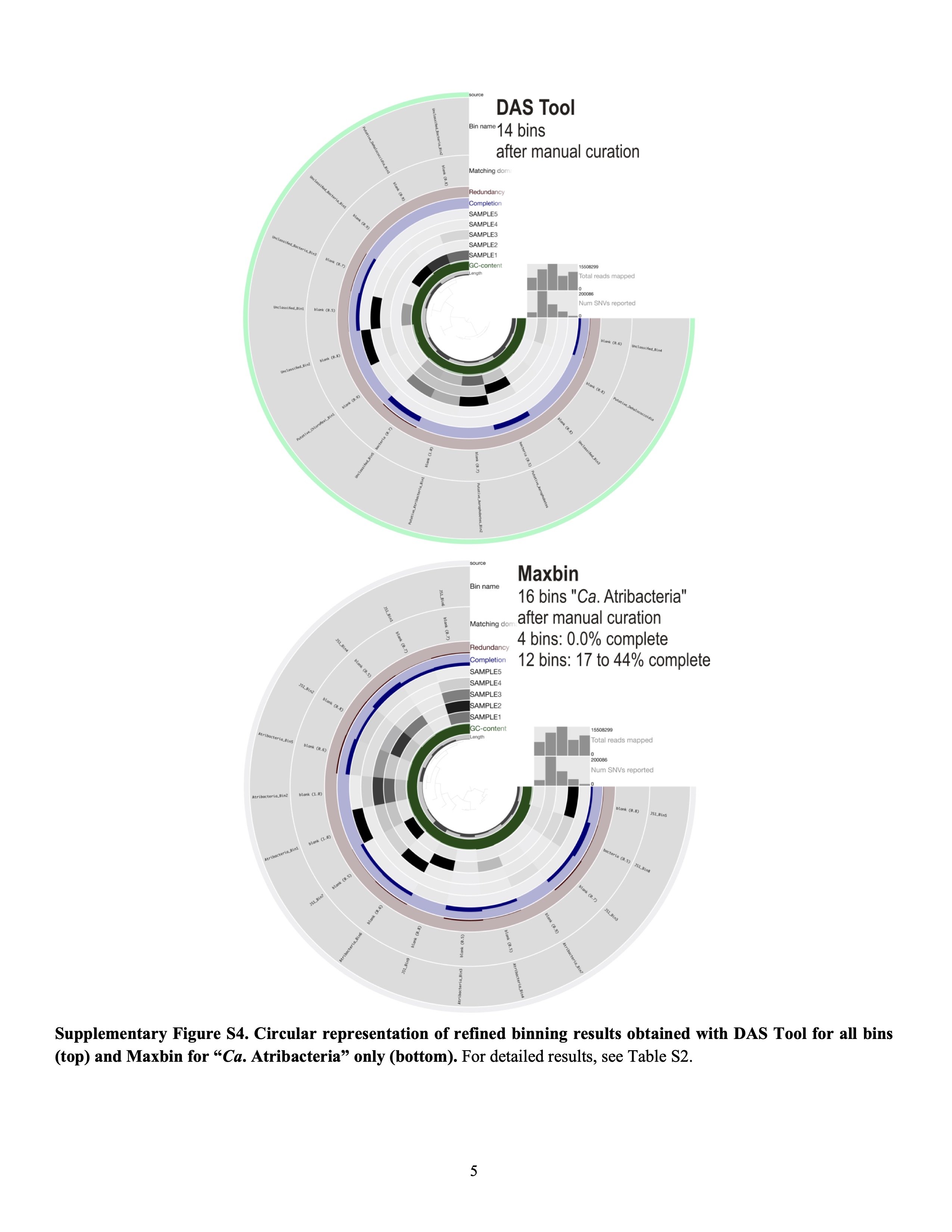

Supplement: FIG S4 [file mBio.01937-20-sf004.jpg]

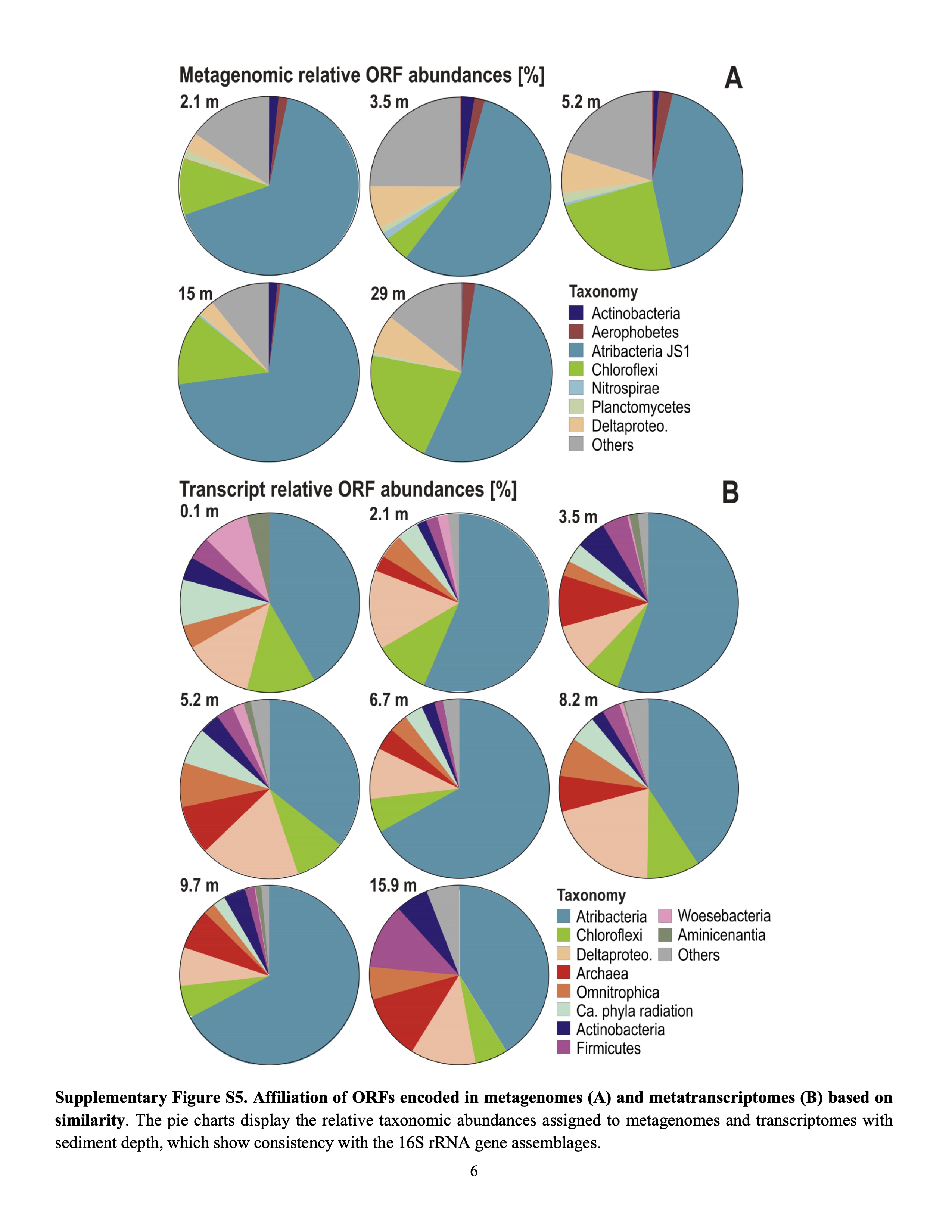

Supplement: FIG S5 [file mBio.01937-20-sf005.jpg]

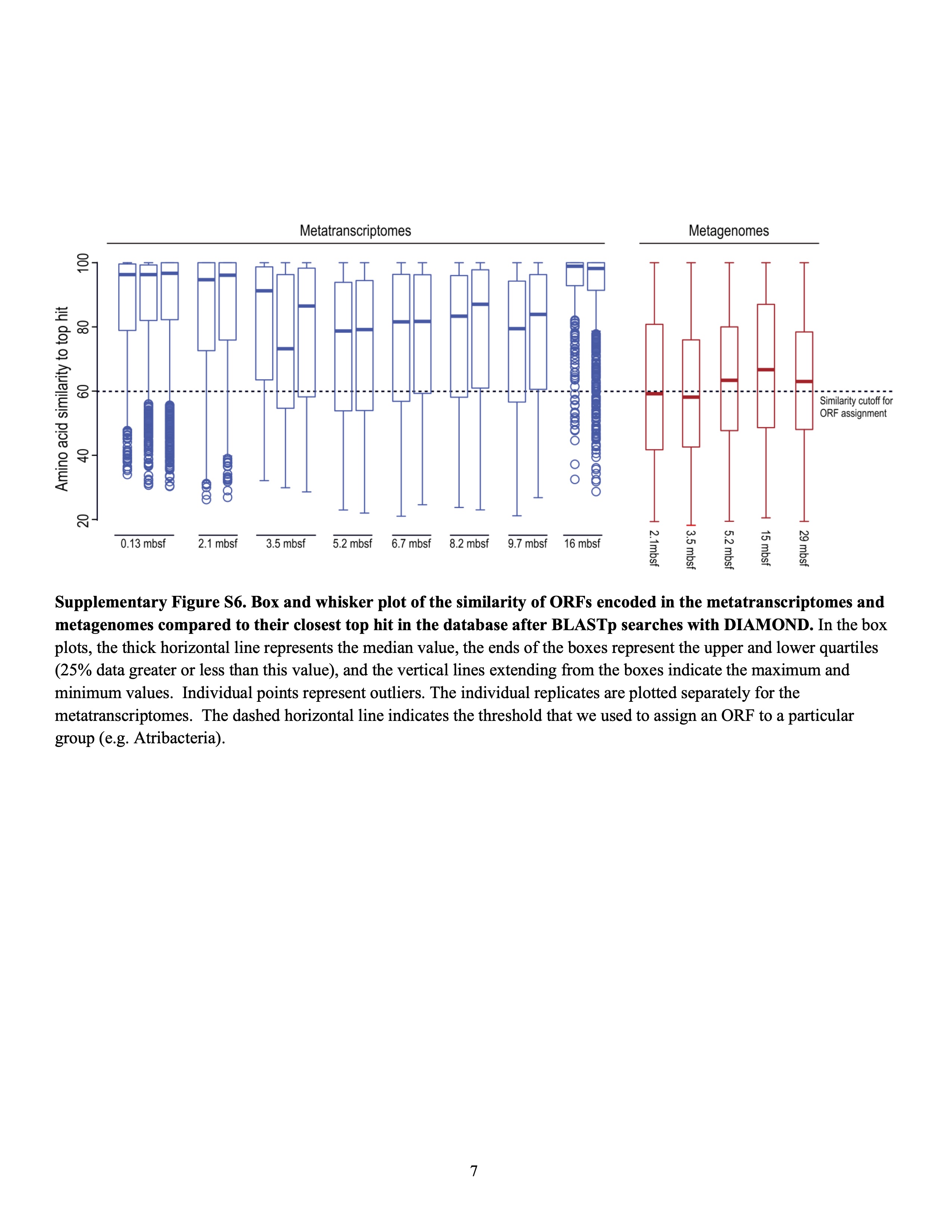

Supplement: FIG S6 [file mBio.01937-20-sf006.jpg]

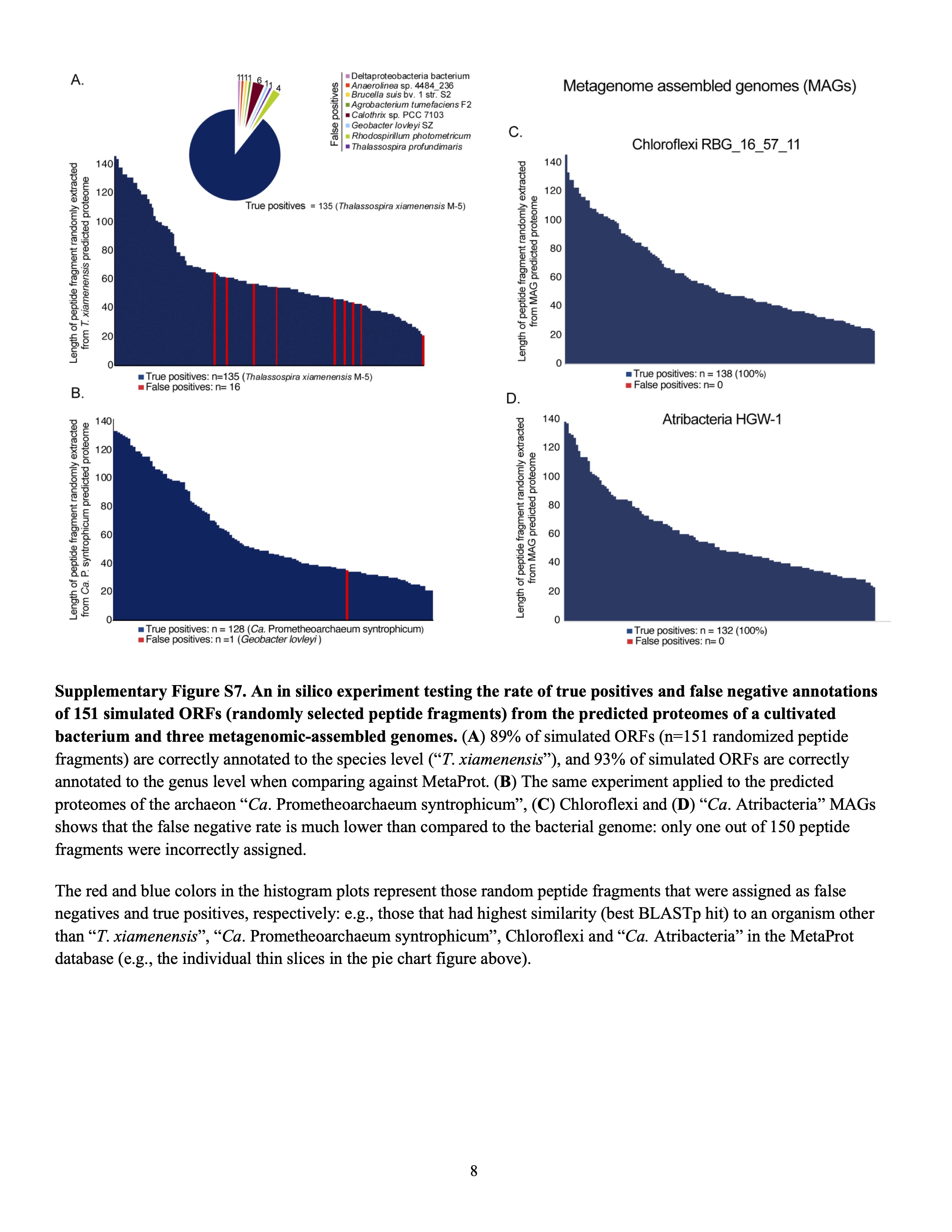

Supplement: FIG S7 [file mBio.01937-20-sf007.jpg]

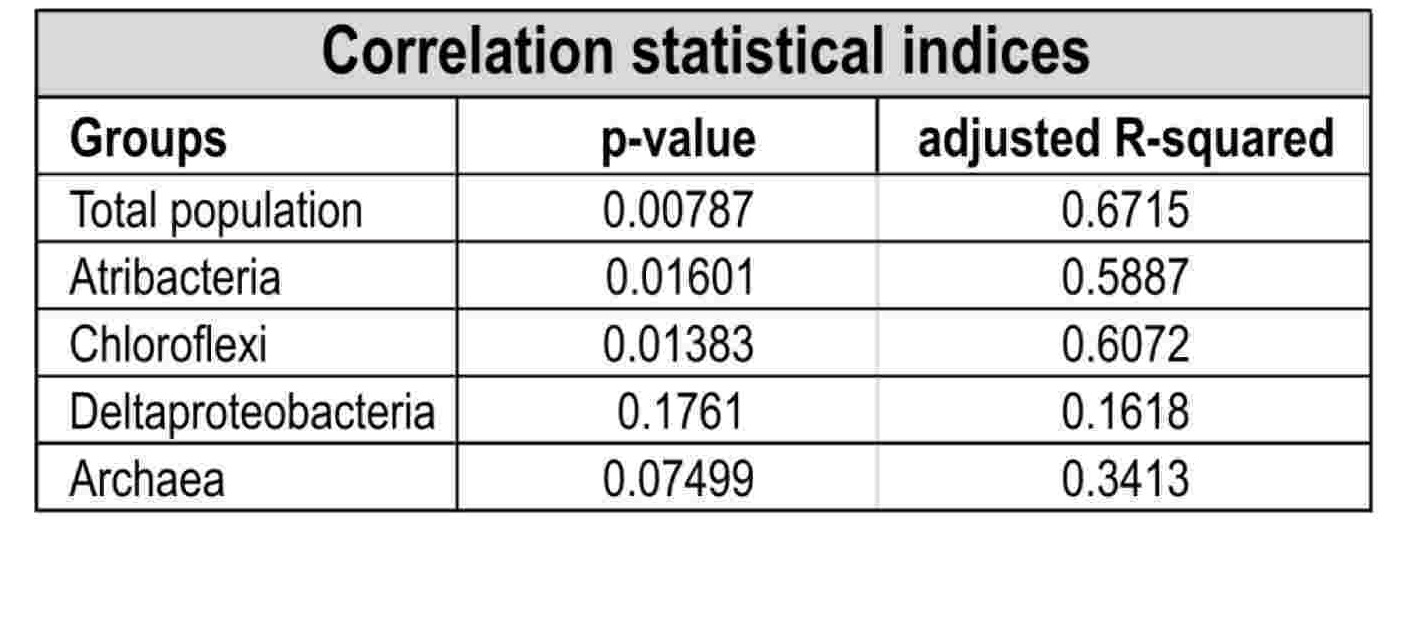

Supplement: TABLE S1 [file mBio.01937-20-st001.jpg]

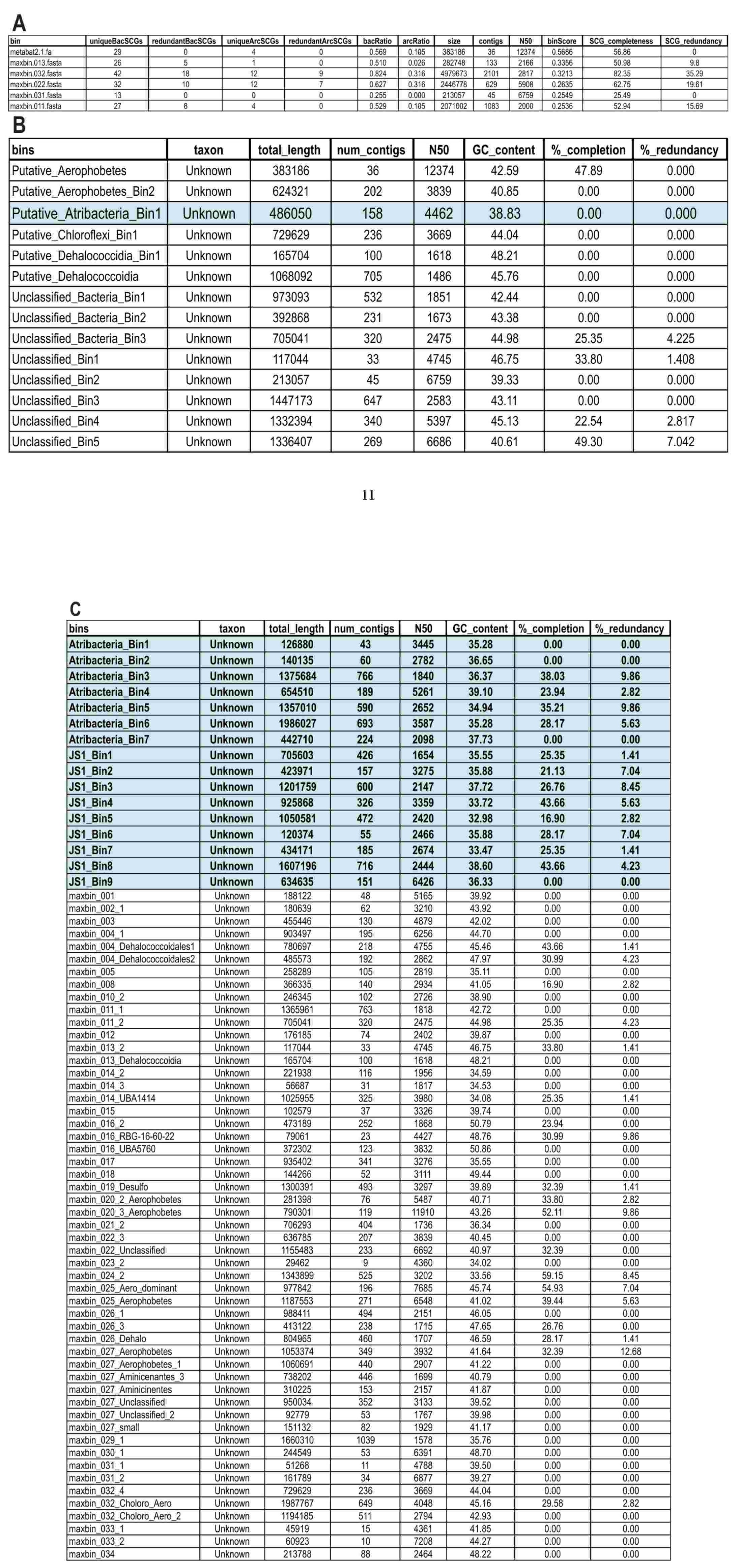

Supplement: TABLE S2 [file mBio.01937-20-st002.jpg]
